# Evaluation of Self-Healing Properties of OPC-Slag Cement Immersed in Seawater Using UPV Measurements

**DOI:** 10.3390/ma16217018

**Published:** 2023-11-02

**Authors:** Choonghyun Kang, Yongmyung Park, Taewan Kim

**Affiliations:** 1 Department of Ocean Civil Engineering, Gyeongsang National University, Tongyeong 53064, Republic of Korea; 2 Department of Civil Engineering, Pusan National University, Busan 43241, Republic of Korea

**Keywords:** slag, ultrasonic pulse velocity, self-healing, seawater, brucite

## Abstract

In this study, OPC-slag cement, which partially replaced ground granulated blast-furnace slag (GGBFS), was immersed in seawater at three temperatures and the self-healing effect was evaluated through ultrasonic pulse velocity (UPV) measurement. In addition, test specimens without cracks were immersed and cured in the same seawater environment to compare the characteristics of UPV and crack-healing effects. The results of the study showed that increasing the GGBFS content or immersion temperature improved the healing effect up to 30 days after immersion, but there was no significant effect after 30 days of immersion. In a saltwater environment, a thick layer of brucite was deposited near the crack, blocking the inflow of seawater and impeding the formation of additional healing material. According to visual observation, the crack entrance appears to have been covered and healed by the brucite layer. However, the brucite layer in the crack area increases the UPV in the early stages of immersion, which may lead to a misconception that it is self-healed, and there is a possibility of overestimating the self-healing effect.

## 1. Introduction

Concrete is one of the most important materials used in the construction of society’s infrastructure. Concrete has various advantages as a construction material, has developed its performance over a long period of time, and is still used in many construction structures. Population growth and urbanization accelerate the construction of infrastructure [1,2]. However, the gradually accelerating environmental pollution and natural disasters reach the limits of the performance of structures, and, in particular, increase the occurrence of cracks, a representative weak point of concrete structures [1,2,3]. Cracks tend to weaken the integrity and bearing capacity of the structure, seriously compromising its safety, usability, and durability. Repair and maintenance of concrete structures is an important means of extending their useful life, but manual maintenance costs are prohibitively high for large-scale infrastructure. In addition, the various types, shapes, exposure environments, crack occurrence locations, widths, and lengths of concrete structures make consistent and systematic maintenance and repair difficult. Therefore, from the maintenance perspective of infrastructure to which concrete is applied, the interest in and the need for self-healing concrete is increasing to reduce time and cost and improve efficiency [2,3,4,5].

Various approaches have been utilized to obtain self-healing concretes [4,5]. For example, capsules inside the concrete structure contain an adhesive solution that restores the cracks automatically [6,7,8,9]; cementitious materials have been used to improve the healing performance of the material itself [10,11,12,13,14,15,16,17,18,19]; bacteria have been used to induce the precipitation of healing substances [20,21,22,23,24]; and expansive agents have been used to seal cracks [25,26,27,28,29,30,31]. Other studies have also examined environmental effects on self-healing, including the presence of mediating substances, moisture, dry–wet cycles, age, healing time, and temperature [6,28,32,33,34,35,36,37]. In previous studies on the self-healing of concrete in fresh water, the rehydration of unhydrated cement particles and the crystallization of healing substances were the principal self-healing mechanisms, with ettringite, C–S–H, calcium hydroxide, and calcium carbonate reported as healing substances [11,26,38,39,40]. Although a few studies have evaluated self-healing in concretes immersed in artificial seawater [14,41,42], little attention has been paid to self-healing in real seawater, despite the fact that real seawater contains various inorganic and organic materials not found in artificial seawater. Artificial seawater reported in previous studies is manufactured with a certain composition ratio of specific components, and it does not reflect the various ions of actual seawater and changes in the composition ratio of ions [14,41]. Artificial seawater is an artificial and ideal environment in which there is no change in the concentration of various ions that make up seawater during the experimental environment and period. Among the methods for studying the properties of concrete in response to changes in the actual seawater environment, mounting or immersion in the actual ocean would be the optimal method, but as a preliminary step, the properties of concrete are analyzed through experiments and research in a laboratory using actual seawater is also needed. In addition to inorganic substances, various microalgae and organisms exist in actual seawater. These organic substances and microalgae grow and live on the surface of concrete structures and affect the long-term durability of concrete. In order to create conditions as similar to the marine environment as possible at the laboratory level, it is believed that conducting experiments using actual seawater rather than artificial seawater will provide more useful data for future experiments and research on actual concrete structures in the ocean. Recently, research is being conducted on self-healing models for various concrete cracks [43].

In recent years, ground granulated blast-furnace slag (GGBFS) or fly ash has been added to concretes to impart salt resistance. Therefore, some studies on concrete self-healing have focused on cements mixed with GGBFS or fly ash [12,44]. The salt resistance imparted by GGBFS means that concrete containing GGBFS can be used to build structures that will be exposed to marine environments. In addition, experimental results on the role and effect of slag to study the effect on self-healing of concrete are also being published [17,45,46]. This study focuses on self-healing in GGBFS-containing concrete immersed in real seawater.

Self-healing properties have been evaluated using a variety of methods [33,34,35]. Tensile testing, three- or four-point bending testing, and acoustic emission analysis are commonly used to measure recovery in mechanical properties, whereas measurements of tightness recovery are often based on water or air permeability, chloride diffusion, or ultrasonic transmission. Appearance-based evaluations can be carried out using scanning electron microscopy (SEM), X-ray tomography, and optical microscopy. Recently, new techniques such as diffuse ultrasound have been applied to the evaluation of self-healing [47]. The measurement of ultrasonic pulse velocity (UPV), a common method that does not damage the specimen and/or the forming healing substances, has also been applied to assess the self-healing effect [10,11,12].

In this study, the self-healing properties of cement specimens partially replaced with different amounts of GGBFS when immersed in real seawater at different temperatures were investigated by measuring ultrasonic velocities. The healing substances and precipitates were observed by SEM, and the chemical composition was confirmed by energy-dispersive X-ray spectroscopy (EDS). In this study, we present a new method to examine the self-healing effect of concrete immersed in seawater by measuring UPV and analyzing the rate of change of UPV. Self-healing effect analysis using the UPV change rate is expected to provide more accurate and clear analysis results along with the various self-healing effect review and analysis methods reported so far.

## 2. Materials and Methods

### 2.1. Materials

The X-ray fluorescence-determined compositions of the ordinary Portland cement (OPC) and GGBFS used in this study along with their physical properties are shown in Table 1.

The experimental specimens were immersed in seawater taken from the southern coast of South Korea. The composition of the experimental seawater was determined using inductively coupled plasma-atomic emission spectrometry, and it is shown in Table 2. The pH of the experimental seawater was measured to be 7.63.

### 2.2. Specimen Preparation

Experimental OPC specimens containing 0%, 10%, 20%, and 30% GGBFS (based on the mass of OPC) were created with water-to-binder ratios of 0.5. The concrete pastes were mixed following ASTM C109, and the specimens were cast in a prismatic steel mold with dimensions of 40 mm × 40 mm × 160 mm. The cast specimens were cured in the chamber at a temperature of 23 ± 2 °C and relative humidity of 85 ± 5%. All specimens were demolded 24 h after casting and then cured in fresh water at a temperature 23 ± 2 °C, for 27 days. 

### 2.3. Healing Experiments

UPV measurements were carried out using a PROSEQ-CCT4 instrument with the direct method. A transmitter and receiver were attached to the opposite ends of the 160 mm side of each specimen. UPV consisted of two parts: a transmitter and a receiver. Initially, the transmitter was on the left side and the receiver was on the right side. Then, the position of transmitter and receiver was reversed, i.e., transmitter was on the right side and receiver was on the left side. An average value of these two measurements was obtained, which was denoted as the average UPV value. The initial UPV (UPV_INI_) of each specimen was measured following curing (day 28) after removing the specimen from fresh water and wiping the wet surface. To induce crack formation, the specimens in the healing group were subjected to three-point bending using a loading of 90% of the average fracture load, as determined in a preliminary experiment [12]. The UPV after crack induction (UPV_CRC_) was then measured. After crack induction, the specimens in the healing group were placed in a polycarbonate mold to prevent further crack propagation during healing and UPV measurement. 

**Figure 1:**
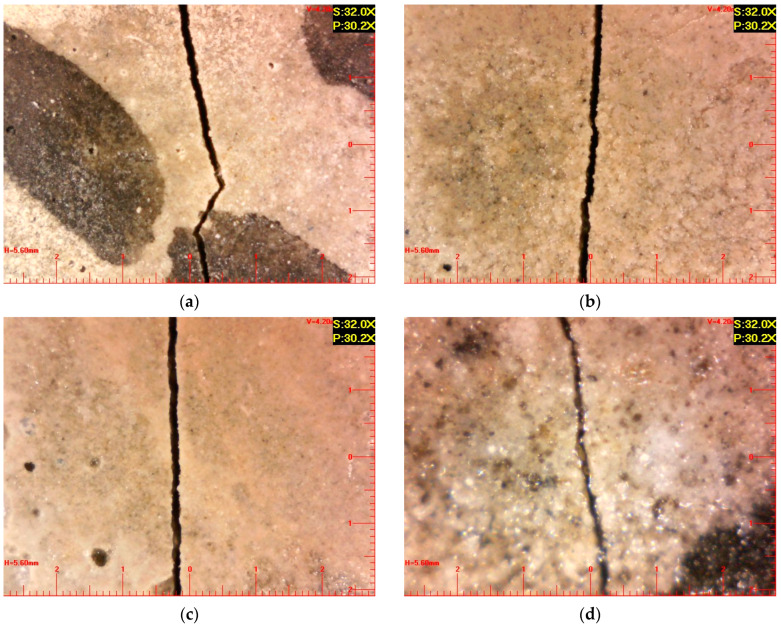
Optical microscopy images of induced cracks in OPC specimens containing different contents of GGBFS: (**a**) 0%, (**b**) 10%, (**c**) 20%, and (**d**) 30%.

In past studies, crack width was identified as a major factor affecting the self-healing of concrete [25,42]. On the basis of these studies, only specimens with crack widths of less than 200 μm were selected for this study, considering the research purpose (self-healing) and methods (UPV, SEM, and EDS). For every experimental condition, three specimens with cracks less than 200 μm in width were obtained. Figure 1 shows example optical microscopy images of cracks induced in specimens with different GGBFS contents.

**Figure 2:**
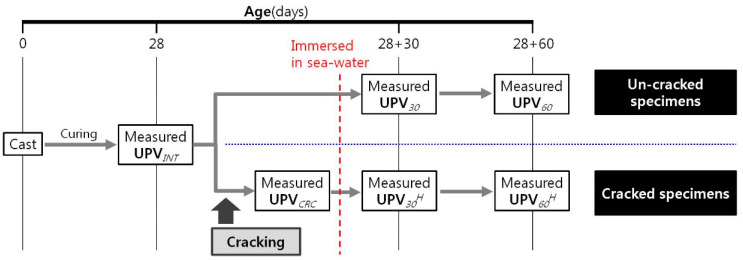
Experimental scheme.

The specimens were immersed in seawater after measuring UPV_CRC_ (for the healing group) or after measuring UPV_INT_ (for the control group). In both groups, the specimens were kept in thermo-hygrostat chambers at three different temperatures: 5 ± 0.2 °C, 15 ± 0.2 °C, and 25 ± 2 °C. Concrete structures placed in the marine environment are exposed to various seawater conditions. Among these, the temperature of sea water varies, depending on the location of the structure and the depth of the water. Since the temperature decreases from the room temperature in areas close to sea level (sea level, tidal zone or splash zone) as the water depth increases, temperature changes up to 5 °C were set considering the water depth of marine concrete structures such as general ports. Therefore, when performing this experiment, three sea temperature conditions of 5, 15, and 25 °C were considered at which concrete was exposed to sea water. Seawater was exchanged each week during the immersion period to provide a constant supply of inorganic materials and prevent the re-absorption of eluted material from the cement paste. The UPV was measured in both the control and healing groups after the specimens had been immersed in seawater for 30 days (UPV_30_ and ${UPV}_{30}^{H}$, respectively) and 60 days (UPV_60_ and ${UPV}_{60}^{H}$, respectively). Figure 2 shows the experimental scheme.

After measuring ${UPV}_{30}^{H}$ and ${UPV}_{60}^{H}$, a small piece that included the crack was cut from each specimen in the healing group. These pieces were immersed in acetone for 48 h to prevent further hydration or healing and then dried for 48 h. The dried pieces were analyzed by SEM to observe the healing substances and by EDS to determine the chemical components of healing substances and precipitates.

## 3. Results

### 3.1. UPV Measurements

The UPV measurements are shown in Table 3 and Table 4. The change in UPV was calculated from the measured UPV values in each table. The initial UPV values were different for the specimens, therefore, it was not easy to compare these values. Thus, the rate of change in UPV was calculated. For the control group, the rates of increase in UPV from after curing to 30 days of immersion and from 30 to 60 days of immersion were, respectively, calculated as

(1) $${∆UPV}_{INT-30}=\frac{{UPV}_{30}-{UPV}_{INT}}{{UPV}_{INT}}\times 100$$

(2) $${∆UPV}_{30–60}=\frac{{UPV}_{60}-{UPV}_{30}}{{UPV}_{30}}\times 100$$

For the healing group, the rates of change in UPV from after crack induction to 30 days of immersion and from 30 to 60 days of immersion were, respectively, calculated as

(3) $${∆UPV}_{CRC-30}^{H}=\frac{{UPV}_{30}^{H}-{UPV}_{CRC}^{H}}{{UPV}_{CRC}^{H}}\times 100$$

(4) $${∆UPV}_{30–60}^{H}=\frac{{UPV}_{60}^{H}-{UPV}_{30}^{H}}{{UPV}_{30}^{H}}\times 100$$

Table 3 shows the measured UPV values of the control specimens. At all three experimental temperatures, UPV_30_ was greater than UPV_INT_, and UPV_60_ was greater than UPV_30_. The increase in UPV with immersion time was attributed to the densification of the microstructure and reduction in capillary volume as hydration progressed; that is, the increase in UPV in the control group was explained by the hydration effect.

Table 4 shows the measured UPV values of the specimens in the healing group. At all tested temperatures, ${UPV}_{CRC}$ was lower than ${UPV}_{INT}$, and UPV then increased with seawater immersion time. These increases in UPV are attributed to two factors: the self-healing effect in the cracked part of the specimen and the hydration effect in the un-cracked part. In the pure OPC specimens (0% GGBFS), ${UPV}_{30}^{H}$ was less than ${UPV}_{INT}$; contrastingly, in the specimens containing more than 10% GGBFS, ${UPV}_{30}^{H}$ was greater than ${UPV}_{INT}$. This difference is attributed to GGBFS hydration, which occurs more slowly than OPC hydration and occurs at exposed parts of the crack surface along with inside the specimen. The effect of un-hydrated raw materials at the exposed parts of the crack surface has been studied using pozzolan materials such as slag and fly ash [12]. Furthermore, the increase in UPV with increasing immersing time reflects the slow hydration of un-hydrated particles inside the specimen [10,32,33,38]. 

Table 3 and Table 4 show the rates of change in UPV, calculated using Equations (1)–(4). In Table 3, the value of ${∆UPV}_{INI-30}$, which represents the hydration effect during the first 30 days of immersion, increased smoothly with increasing GGBFS content. It should be noted that the slow hydration effect of GGBFS is more prevalent than the initial hydration effect of OPC in ${∆UPV}_{INI-30}$ because ${UPV}_{INT}$ was measured 28 days after casting, and the slow hydration effect is proportional to the GGBFS replacement ratio. The same trend was observed in ${∆UPV}_{30–60}$, the UPV increased with an increase in the GGBFS content. In the GGBFS replacement specimen, the rates of UPV from 30 to 60 days were higher than the rates of UPV from the initial curing (${UPV}_{INT}$) to 30 days (${∆UPV}_{30–60}$ > ${∆UPV}_{INT-30}$). However, the pure OPC specimen (0% GGBFS) exhibited the lowest rate of change in UPV during both immersion periods, and ${∆UPV}_{30–60}$ was smaller than ${∆UPV}_{INT-30}$. This indicates that the hydration effect of OPC without any pozzolan material continues to decrease. Regardless of the GGBFS content and the immersion period, immersion temperature was positively associated with the rate of increase in UPV. This is consistent with a previous study in which increasing immersion temperature promoted the hydration of un-hydrated particles [36]. In summary, the rate of change in UPV increased with increasing GGBFS content and immersion temperature, and the rate also increased slightly from the first 30 days of immersion to the second 30 days, with the exception of the sample with 0% GGBFS.

The ΔUPV values in the healing group showed the same trends as those in the control group (Table 4); ΔUPV increased with increasing GGBFS content, which might also be explained by slow hydration. Immersion temperature was positively associated with ΔUPV in both immersion periods, although the effect of the immersion temperature was more significant in the first period (${∆UPV}_{CRC-30}^{H}$) compared to the second period (${∆UPV}_{30–60}^{H}$). There is, however, a significant gap between ${∆UPV}_{CRC-30}^{H}$ and ${∆UPV}_{30–60}^{H}$. ${∆UPV}_{CRC-30}^{H}$ were relatively higher than ${∆UPV}_{INT-30}$, and ${∆UPV}_{30–60}^{H}$ were slightly lower than ${∆UPV}_{30–60}$. In the healing group, reactions occurred within the specimens and on the cracked surfaces (i.e., self-healing reactions). During the immersion period, the un-hydrated particles in OPC-GGBFS gradually undergo a rehydration reaction, affecting self-healing [13,39,48]. Additionally, in this study, the slow hydrated substance of GGBFS exposed to the cracked surface could also be considered. Therefore, the high ${∆UPV}_{CRC-30}^{H}$ indicates that the self-healing reaction primarily occurred during the first 30 days of immersion after cracking, which is consistent with previous studies [39,47,49]. 

Because the ultrasonic pulse passes through both the un-cracked, cracked and healed parts of the sample, the measured UPV values include the effects of both hydration and self-healing. Although it may not be possible to isolate the self-healing effect, the following assumptions were made to eliminate the hydration effect as completely as possible: (1) within the same immersion period, the hydration effect in the control group was assumed to be equal to that in the healing group, and (2) ΔUPV in the healing group was assumed to represent the arithmetic sum of the hydration and self-healing effects. On the basis of these assumptions, the purely self-healing effects in the first and second 30 day periods of immersion can be, respectively, calculated as arithmetic differences:

(5) $${∆SHE}_{CRC-30}={∆UPV}_{CRC-30}^{H}-{∆UPV}_{INI-30}$$

(6) $${∆SHE}_{30–60}={∆UPV}_{30–60}^{H}-{∆UPV}_{30–60}$$

where ${∆SHE}_{CRC-30}$ represents the self-healing effect in the first immersion period (from cracking to 30 days of immersion) and ${∆SHE}_{30–60}$ indicates the self-healing effect in the second immersion period (from 30 to 60 days of immersion). A positive ∆SHE indicates that the self-healing effect is stronger than the hydration effect (i.e., the increase in UPV can be attributed primarily to self-healing). Conversely, negative ∆SHE indicates that the increase in UPV can be explained primarily by the hydration effect.

**Figure 3:**
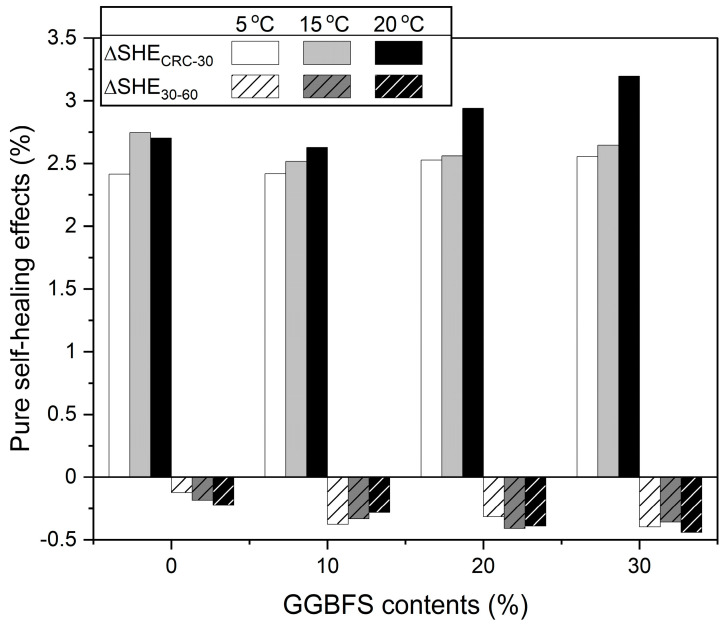
Effects of self-healing only in the two periods of immersion (${∆SHE}_{CRC-30}$: No hatch pattern, and ${∆SHE}_{30-60}$: There is a hatch pattern).

Figure 3 shows the values of ∆SHE calculated using Equations (5) and (6). In Figure 3, ${∆SHE}_{CRC-30}$ has no hatch pattern and is expressed as positive values above 0, while ${∆SHE}_{CRC-30}$ has a hatch pattern and is expressed as negative values below 0. ${∆SHE}_{CRC-30}$ showed positive values ranging from 2.41 to 3.19, indicating that the self-healing effect was dominant during the first 30 days after immersion. When the GGBFS content was 10–30%, the self-healing effect became stronger as immersion temperature increased from 5 °C to 25 °C, and the self-healing effect increased slightly with increasing GGBFS content. However, the 0% GGBFS replacement case did not show consistent change according to temperature, and those were not also the smallest at the same temperature. The following conclusions were drawn from the above results. The self-healing effect in concrete specimens containing GGBFS was dependent on the rehydration of GGBFS particles exposed at cracks, and the self-healing effect became stronger with increasing GGBFS content and immersion temperature. Moreover, immersion temperature had a stronger effect on self-healing than GGBFS content in this study. The overall temperature is relatively low (5–25 °C) and the temperature difference was not as high as 10 °C; therefore, apparent difference [36] was not observed. The different trend of 0% GGBFS cases (100% OPC) was considered because of the absence of GGBFS.

All values of ${∆SHE}_{30–60}$ were negative and were not significantly altered by GGBFS content or immersion temperature. This indicates that self-healing was no longer dominant during the second 30 days of seawater immersion. Thus, the increase in UPV in this period resulted primarily from the hydration effect. 

### 3.2. SEM and EDS Analyses

**Figure 4:**
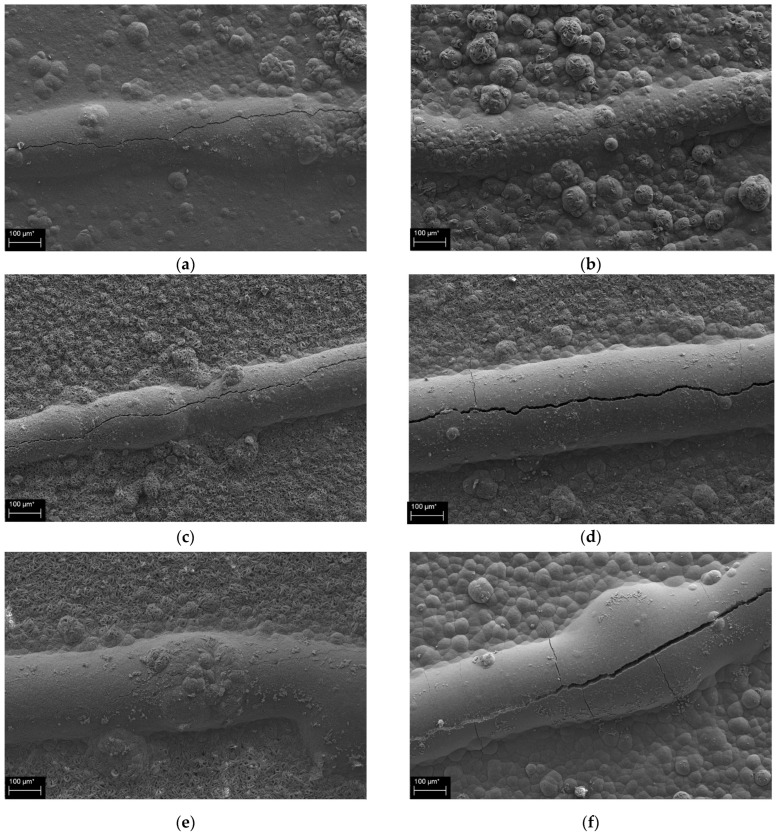
SEM images of the cracked parts of the specimens with different GGBFS contents and immersion temperatures: (**a**) 0% GGBFS, 5 °C, (**b**) 10% GGBFS, 5 °C, (**c**) 10% GGBFS, 15 °C, (**d**) 30% GGBFS, 15 °C, (**e**) 10% GGBFS, 25 °C, and (**f**) 30% GGBFS, 25 °C.

Figure 4 shows representative SEM images of the cracked parts of the specimens in the healing group after 60 days of immersion for different GGBFS contents and immersion temperatures. In each case, precipitates were confirmed on the entire area of observation. The sizes and shapes of the precipitates varied, and thick precipitate layers were identified along the cracks. The crack shown in the thick layer of some SEM images occurred during the dry sequence of the precipitation process of SEM sample. The observed precipitates were assumed to be composed of inorganic ions from seawater.

**Figure 5:**
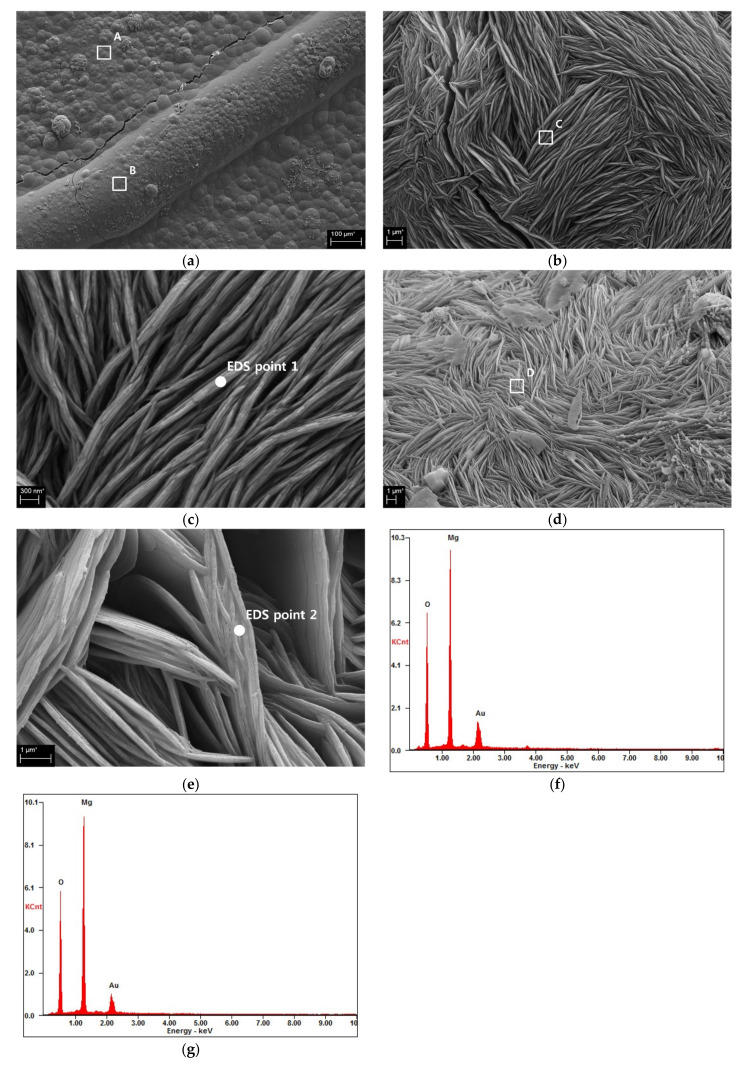
SEM images and EDS spectra of the precipitates formed around and along the crack in a specimen containing 20% GGBFS and immersed in seawater at 15 °C: (**a**) 20% GGBFS, 15 °C, (**b**) enlarged view of location A, (**c**) enlarged view of location C, (**d**) enlarged view of location B, (**e**) enlarged view of location D, (**f**) EDS spectrum of EDS point 1, and (**g**) EDS spectrum of EDS point 2.

Figure 5 shows SEM images with different magnifications of specific locations along with the EDS results for a specimen containing 20% GGBFS and immersed in seawater at 15 °C. Two representative locations were selected: a precipitate formed around the crack (location A in Figure 5a) and a thick precipitate formed along the crack (location B in Figure 5a). 

The precipitate around the crack formed irregularly shaped masses around location A in Figure 5a. Figure 5b, which depicts an enlarged view of location A, shows thin and long needle-shaped materials piled up in several directions. Figure 5c, which shows a further enlarged image, reveals a thin, plate-like shape with a thickness of approximately 200 nm. The EDS results from the points shown in Figure 5c indicate that the precipitate at location A was brucite (Figure 5f). The overall shapes of the precipitates shown in Figure 5d,e, which are enlarged views of location B, are similar to the images of location A; however, the precipitate formed at location A was slightly denser than that formed in location B (compare Figure 5c,e). The EDS spectrum in Figure 5g indicates that the precipitate at location B was also brucite. 

Magnesium precipitates have been identified on the surfaces of concrete structures immersed in seawater in numerous studies, primarily as a result of the magnesium ions in seawater [14,50,51,52,53]. Brucite and aragonite have also been observed on the surfaces of cracked specimens immersed in seawater and fresh water, respectively [41]. Furthermore, brucite was found to seal cracks in specimens immersed in artificial seawater [14]. The precipitation of brucite in this study is consistent with the previously reported results. 

**Figure 6:**
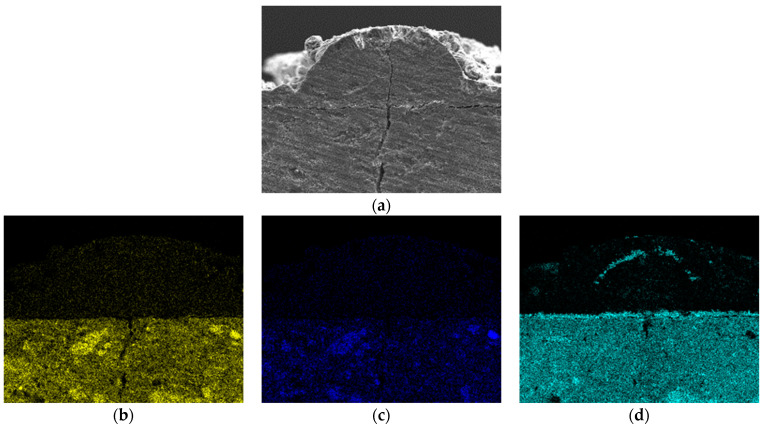

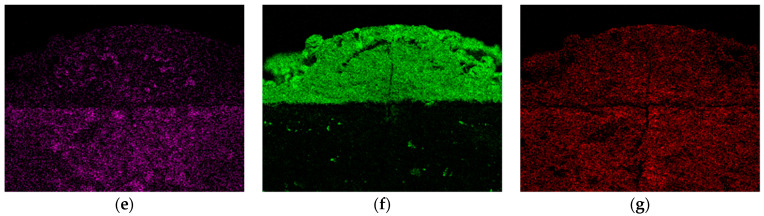
(**a**) SEM image and (**b**–**g**) EDS elemental maps for the vertical cross section in the direction of crack propagation: (**a**) Crack mouth, (**b**) Si (silicon), (**c**) Al (aluminum), (**d**) Ca (calcium), (**e**) K (potassium), (**f**) Mg (magnesium), and (**g**) Na (sodium).

Figure 6 shows the SEM image and EDS elemental maps of the vertical cross section in the direction of crack propagation, confirming the thickness and composition of the precipitate. The main component of the precipitate covering the crack was brucite (Figure 6f). Brucite was evenly deposited on the surface with considerable thickness and constituted the entire thick layer along the crack. Calcium (Figure 6d), potassium (Figure 6e), and sodium (Figure 6g) were detected in the seawater. These findings support the SEM and EDS results.

**Figure 7:**
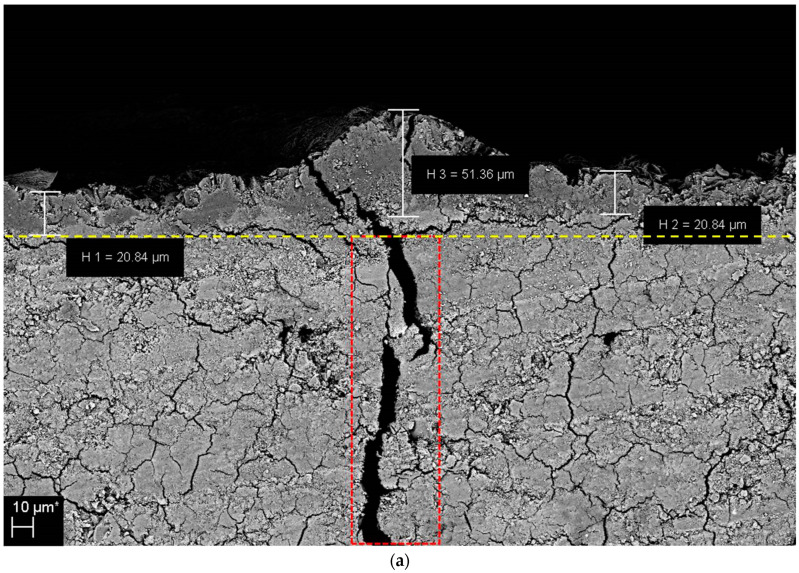

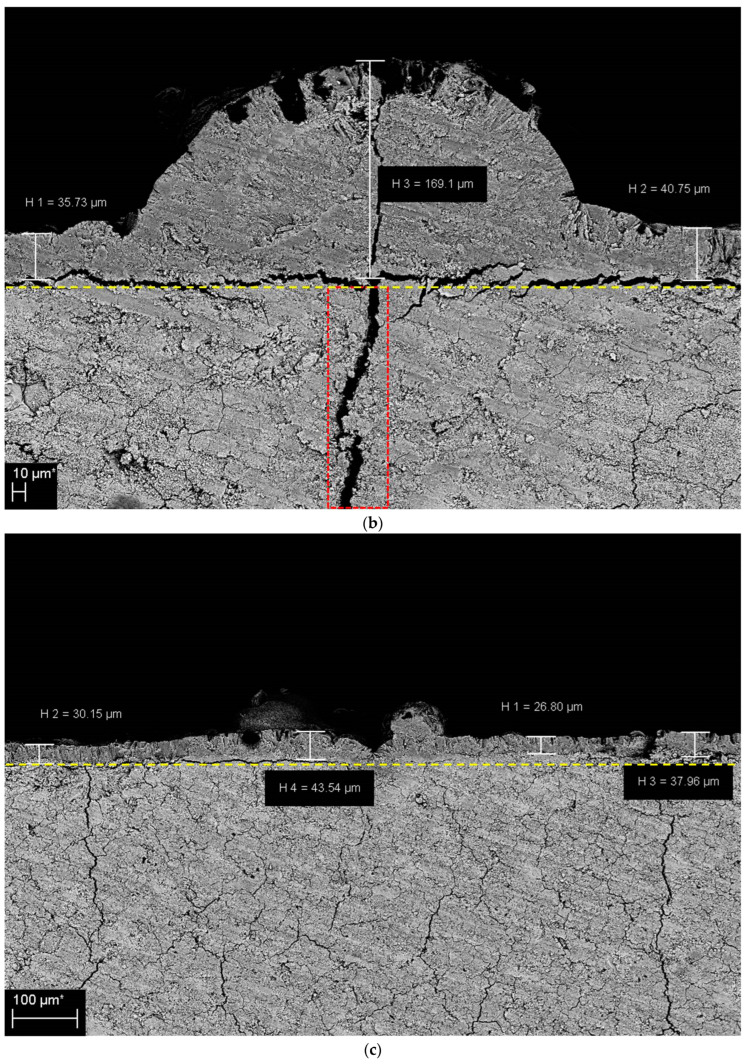
SEM analysis of precipitate thickness at the mouth of the crack and sample surface: (**a**) Crack mouth (20% GGBFS, 25 °C, 30 days), (**b**) Crack mouth (20% GGBFS, 25 °C, 60 days), and (**c**) Surface (30% GGBFS, 15 °C, 60 days) (Yellow dotted line: interface between specimen and precipitated brushite, Red dotted box: cracks artificially created in the sample by applying load).

Figure 7a,b show the SEM-analyzed thicknesses of the precipitated brucite layer for the sample containing 20% GGBFS and immersed in seawater at 25 °C for 30 and 60 days, respectively. The red box indicates the crack induced in the sample by loading. The yellow dashed line indicates cracks that occurred at the interface between the specimen and precipitated brucite; these cracks were presumed to have been generated when the sample was dried for SEM observation. After 30 days of immersion (Figure 7a), the thickness of the brucite layer along the crack was approximately 51.36 μm, and the thickness of the flat layer around the crack was approximately 20.84 μm; after 60 days, the corresponding values were approximately 169.1 and 35.73–40.75 μm, respectively (Figure 7b). Figure 7c indicates that the thickness of the flat precipitate far from the crack was approximately 26.80–43.54 μm. Although the thickness of the precipitate varied from sample to sample, the overall trends were consistent: the thick layer and the flat layer became thicker with increasing immersion time, and both of these layers were thicker than the flat layer far from the crack.

As shown in Figure 7, the thickness of the precipitate along the crack observed in Figure 4 was two to five times greater than the thickness of the precipitates in other regions. This is likely attributed to the large contact area with seawater in the area around the crack. A comparison of Figure 7a,b suggests that the thickness of the brucite layer along the crack increased with immersion time. In contrast, the brucite thickness was not significantly affected by immersion temperature in the range of 5 °C to 25 °C or GGBFS content. 

**Figure 8:**
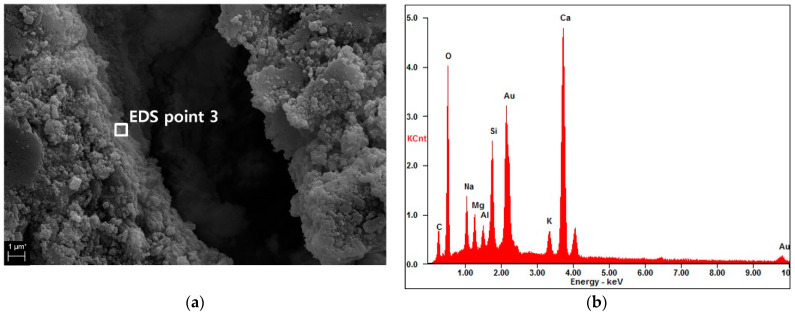

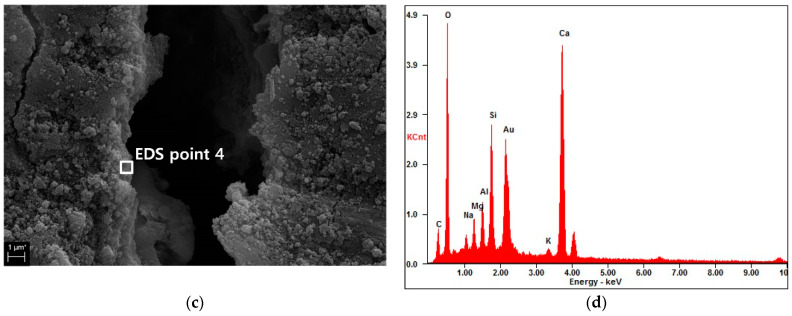
(**a**,**c**) SEM images and (**b**,**d**) corresponding EDS patterns of the crack surfaces indicated by the red boxes in Figure 8a,b: (**a**) Crack surface (10% GGBFS, 25 °C), (**b**) EDS pattern of EDS point 3 in (**a**), (**c**) Crack surface (20% GGBFS, 5 °C), and (**d**) EDS pattern of EDS point 4 in (**c**).

Figure 8 shows the SEM images and the corresponding EDS spectra of the inside of the crack induced by loading (indicated by the red box in Figure 7) in the specimen with 20% GGBFS and immersed in seawater at 25 °C. Despite the presence of precipitates with thicknesses of 20~160 μm around the outside of the crack, no chemical precipitates were observed on the crack surfaces. The EDS analysis results in Figure 8b,d show that hydration reactants presumed to be CaCO_3_, C–S–H and ettringite were found on the internal crack surface. These hydration reactants are similar to the EDS analysis results reported by Sisomphon et al. [26]. The hydration reaction observed on the internal crack surface did not completely close the crack, and the crack surfaces were still separated from each other. Unlike near the crack entrance, it is believed that significant hydration and soaking time will be required to fill the internal crack. Brucite was also detected, but it was not a major constituent as in the precipitate that formed outside of the crack. These results are consistent with the elemental maps shown in Figure 6. Within the area of observation, no crack surfaces were completely closed or sealed. In this study, the formation of self-healing products required a continuous supply of inorganic materials from the seawater. However, the crack inlet, which served as the only inflow of additional materials from the seawater, was blocked by brucite precipitates after less than 30 days of immersion (Figure 7). This may explain why self-healing products other than brucite were not well formed in this study.

### 3.3. Discussion

As expected for a self-healing material, the UPV value increased rapidly during the first 30 days after immersion. The increase in UPV during this first immersion period may have been caused by the sealing of micro-cracks and the precipitation of brucite at the crack entrance. The effect of brucite was expected to be pronounced in the first immersion period as brucite has been shown to precipitate in time periods as short as 14 days [27,41]. However, the increase in UPV from 30 to 60 days of immersion was extremely low. This can be attributed to the blocking of the crack entrance by the precipitated brucite, which prevented a continuous supply of inorganic materials from seawater and thus limited the production of healing materials inside the crack. In addition, the slow hydration of GGBFS requires calcium hydroxide; however, most calcium hydroxides were depleted in the production of brucite. Thus, little calcite, C–S–H, or other non-brucite precipitates were formed by the hydration of GGBFS during this period. 

The SEM and EDS analyses presented above confirm that a significant brucite precipitation occurred at the crack entrances as a result of the chemical reaction with seawater as follows [42]. Upon immersion, seawater (pH ≒ 7.5) penetrates into the crack in the specimen and contacts the cement paste (pH ≒ 13). The matrix of the cement paste then elutes calcium ions into the seawater [27,41]. In particular, the calcium hydroxide in the cement paste dissolves ($K_{sp}=4.1\times 10^{-6}$) [26] and reacts with magnesium ions in the seawater, as shown in Equations (7)–(9):

(7) $$\begin{array}{c} {\mathrm{MgSO}}_{4}+{\mathrm{Ca}(\mathrm{OH})}_{2}\to {\mathrm{Mg}(\mathrm{OH})}_{2}+{\mathrm{CaSO}}_{4}\cdot {2H}_{2}O\\ \;\mathrm{brucite}\;\mathrm{gypsum}\; \end{array}$$

(8) $$\begin{array}{c} {\mathrm{MgSO}}_{4}+[\;{\mathrm{Ca}(\mathrm{OH})}_{2}+\mathrm{CaO}\cdot {\mathrm{Al}}_{2}O_{3}\cdot {\mathrm{CaSO}}_{4}\cdot {18H}_{2}O]\;\to {\mathrm{Mg}(\mathrm{OH})}_{2}+3\mathrm{CaO}\cdot 3\mathrm{CaO}{\cdot \mathrm{Al}}_{2}O_{3}\cdot {3\mathrm{CaSO}}_{4}\cdot {32H}_{2}O\\ \;\mathrm{calciummonosulfoaluminatehydrate}\;\mathrm{brucite}\;\mathrm{ettringite}\; \end{array}$$

(9) $$\begin{array}{c} {\mathrm{MgCl}}_{2}+{\mathrm{Ca}(\mathrm{OH})}_{2}\to {\mathrm{Mg}(\mathrm{OH})}_{2}+{\mathrm{CaCl}}_{2}\\ \;\mathrm{brucite}\;\mathrm{calciumchloride} \end{array}$$

Because brucite has low solubility ($K_{sp}=1.2\times 10^{-11}$) [48], it precipitates rapidly. At the same time, a portion of calcium hydroxide elutes from the cement paste and reacts with the carbonates in seawater to form calcium carbonate via Equation (10):

(10) $$\begin{array}{c} {\mathrm{CO}}_{2}+{\mathrm{Ca}(\mathrm{OH})}_{2}\to {\mathrm{CaCO}}_{3}+H_{2}O\\ \;\mathrm{calciumcarbonate} \end{array}$$

According to a report by Wang et al. [53], when a test specimen is immersed in seawater, a brucite layer is formed on the surface of the test specimen, and this becomes denser and thicker as the immersion period increases. This is because a large amount of magnesium ions are supplied from seawater. As a result, it was mentioned that the concentration of Mg^2+^ and OH^−^ increased near the crack mouth and precipitated as brucite. Additionally, a very small amount of calcium carbonate or calcium hydroxide was observed, and it was observed at a relatively insignificant level compared to brucite. A similar phenomenon was observed in the experiment of Wang et al. [53] mentioned above. Magnesium ions are known to interfere with the precipitation of calcite [54]. Carbonates and magnesium ions generate a concentration gradient between the crack surface in the cement specimen and seawater. This concentration gradient moves along the crack until the calcium and hydroxide ions in the cement paste matrix meet the magnesium and carbonate ions in the seawater; the ions thus become concentrated at the crack entrance, where the precipitates are formed. A study by Palin et al. [42] also reported an analysis of this phenomenon as follows. The formation of brucite continues until calcium hydroxide is no longer eluted from the cement paste. The cement paste matrix is able to supply more calcium and hydroxide ions as the area of the crack surface increases; thus, the degree of self-healing via brucite precipitation depends on the crack width and the concentration of ions in contact with the crack surface.

Accordingly, the SEM and EDS analyses indicated that little calcium or magnesium was located on the insides of the crack surfaces, and rehydrated or hydrated GGBFS particles were not observed. Consequently, the self-healing effect was dominant in the first 30 days after immersion, whereas the hydration effect was dominant from days 30 to 60. 

The most important finding of this study is that the precipitation of brucite at the crack entrance can result in the overestimation of the self-healing effect. On the basis of visual inspection, precipitation at the crack entrance would likely be assessed as a positive indicator of self-healing. The precipitation of brucite at the crack entrance covers the crack entrance and shortens the ultrasonic transmission path, making it misleading to believe that the crack has healed. Also, during visual inspection, as explained in Figure 5, the crack entrance is completely covered by the precipitation of brucite, and no crack gap or width is observed, so it can be judged to be healed. However, these precipitates can actually restrict the formation of additional healing substances by blocking seawater. Thus, precipitates formed in the crack entrance may need to be considered as a factor that hinders self-healing. Additionally, this study targeted OPC-slag paste. In other words, for mortar or concrete using aggregate, the additional impact of the presence of aggregate on self-healing and UPV needs to be considered. This is something that needs to be further considered in follow-up studies. In addition, it is necessary to expand application to various sizes and shapes of test specimens. This study, which used UPV to measure and analyze the self-healing effect of concrete immersed in seawater, presented sufficiently new perspectives and results, but additional research is needed to address the limitations in the areas mentioned above.

## 4. Conclusions

This study investigated the self-healing effect of OPC specimens with different GGBFS contents immersed in seawater at different temperatures. The healing and hydration effects were evaluated by UPV measurement at 30 and 60 days after immersion, and SEM and EDS analyses confirmed the shapes, sizes, and chemical compositions of the precipitates in and around induced cracks. The key findings are summarized as follows:

The UPV of specimens immersed in seawater increased as immersion time increased, regardless of immersion temperature and GGBFS content. In the control group (no cracks), the increase in UPV was explained by the hydration effect, and in the healed group with cracks, both the hydration effect and self-healing contributed to the increase in UPV. Estimation and analysis of the self-healing effect using the UPV change rate showed quite significant results. Analysis of the UPV change rate showed that the increase in UPV during the first 30 days of flooding was much higher than the increase from days 30 to 60. This means that the self-healing effect was dominant for the first 30 days after immersion, while the hydration effect was dominant from 30 to 60 days. Among the effects on UPV change rate, the healing effect became stronger but not evident as GGBFS content and immersion temperature increased during the first 30 days of immersion. Additionally, no significant correlation was found between 30 and 60 days. The self-healing effect of cracks was confirmed through the UPV change rate, and the main causes of UPV change were analyzed through observation of the crack entrance. SEM and EDS analysis results showed that the precipitate formed around the crack entrance was composed of brucite. Compared to other areas, the thickness of sediments along the cracks was 2 to 5 times thicker. However, no chemical deposits were found inside the crack surface. This means that the brucite precipitated at the crack entrance hindered the continuous supply of inorganic ions from seawater, thereby limiting the production of healing materials inside the crack. Therefore, brucite formation at the crack entrance may result in an overestimation of the self-healing effect, even if it increases the UPV at the beginning of soaking. 

In this study, we proposed a method to separately evaluate the hydration effect and self-healing effect by calculating the UPV change rate for control (no cracks) and cracked specimens. The proposed method is judged to be a method that can distinguish between the hydration effect and the self-healing effect and clearly compare and analyze the self-healing effect. Nevertheless, among the methods of examining the self-healing effect in a seawater environment, applying the change rate of UPV makes it difficult to evaluate the healing effect due to the crack entrance precipitation effect by brucite. Therefore, it is believed that it will be possible to monitor the self-healing status more clearly and accurately by combining other self-healing effect evaluation methods with the UPV change rate.

## Figures and Tables

**Table 1:** Properties of GGBFS and OPC.

		GGBFS	OPC
Chemical Components (%)	SiO_2_	36.05	20.82
	Al_2_O_3_	11.83	4.77
	Fe_2_O_3_	0.82	3.34
	CaO	42.17	62.25
	MgO	3.62	4.03
	TiO_2_	0.74	
	MnO	0.16	
	SO_3_	4.26	3.51
	K_2_O	0.31	0.58
Physical properties	LOI (%)	0.89	1.04
	Blain (m^2^/kg)	420	330
	Density (g/cm^3^)	2.97	3.15

**Table 2:** Component of the experimental seawater (ppm).

	Ca^2+^	K^+^	Mg^2+^	Na^+^	Cl^−^	SO_4_^2−^
Seawater	420 ± 20	470 ± 40	1380 ± 60	8700 ± 130	22,500 ± 80	2760 ± 70

**Table 3:** Measured UPV values in the control group (without cracks).

		UPV (m/s) (Standard Deviation)				
GGBFS Content	Seawater Temperature (°C)	UPV before Loading (${UPV}_{INT}$)	After 30 Days (${UPV}_{30}$)	After 60 Days (${UPV}_{60}$)	ΔUPV_INT-30_	ΔUPV_30–60_
0%	5	4002.1 (0.33)	4031.0 (0.27)	4055.5 (0.52)	0.722	0.608
	15	4010.3 (0.27)	4040.2 (0.31)	4068.8 (0.47)	0.746	0.708
	25	4007.5 (0.41)	4043.1 (0.24)	4077.1 (0.46)	0.888	0.841
10%	5	3978.0 (0.32)	4012.0 (0.29)	4048.0 (0.43)	0.855	0.897
	15	3980.2 (0.39)	4018.1 (0.35)	4059.6 (0.51)	0.952	1.033
	25	3985.4 (0.41)	4027.1 (0.44)	4073.1 (0.62)	1.046	1.142
20%	5	3965.4 (0.33)	4010.2 (0.30)	4058.2 (0.73)	1.13	1.197
	15	3965.9 (0.44)	4013.0 (0.34)	4067.1 (0.68)	1.188	1.348
	25	3960.5 (0.29)	4010.0 (0.25)	4068.3 (0.69)	1.25	1.454
30%	5	3945.9 (0.28)	3992.1 (0.36)	4047.3 (0.53)	1.171	1.383
	15	3946.2 (0.31)	3992.1 (0.27)	4055.1 (0.66)	1.287	1.455
	25	3945.9 (0.35)	4000.0 (0.43)	4065.8 (0.61)	1.371	1.645

**Table 4:** Measured UPV values in the healing group (with cracks).

		UPV (m/s) (Standard Deviation)					
GGBFS Content	Seawater Temperature (°C)	UPV before Loading (${UPV}_{INT}$)	After Loading (${UPV}_{CRC}$)	After 30 Days (${UPV}_{30}^{H}$)	After 60 Days (${UPV}_{60}^{H}$)	${\Delta UPV}_{CRC–30}^{H}$	${\Delta UPV}_{30–60}^{H}$
0%	5	4001.2 (0.27)	3871.2 (0.96)	3992.7 (1.11)	4012.0 (1.23)	3.139	0.483
	15	4005.6 (0.23)	3872.3 (0.87)	4007.5 (0.95)	4028.5 (1.17)	3.491	0.524
	25	4009.1 (0.30)	3874.1 (1.05)	4013.2 (1.20)	4038.0 (1.21)	3.591	0.618
10%	5	3979.0 (0.31)	3855.0 (1.13)	3981.2 (1.18)	4002.0 (1.14)	3.274	0.522
	15	3983.2 (0.35)	3864.0 (0.98)	3998.0 (1.21)	4026.0 (1.28)	3.468	0.701
	25	3981.4 (0.29)	3857.5 (1.26)	3998.7 (0.99)	4033.1 (1.15)	3.674	0.86
20%	5	3961.0 (0.36)	3828.0 (1.17)	3968.0 (1.07)	4003.0 (1.24)	3.657	0.882
	15	3962.3 (0.32)	3825.0 (1.09)	3968.3 (1.14)	4005.6 (1.29)	3.746	0.94
	25	3963.0 (0.38)	3830.5 (1.14)	3991.0 (1.16)	4033.5 (1.22)	4.19	1.065
30%	5	3946.5 (0.31)	3811.0 (1.26)	3953.8 (1.10)	3992.0 (1.28)	3.726	0.987
	15	3947.1 (0.29)	3815.3 (1.17)	3965.1 (1.25)	4008.5 (1.20)	3.934	1.095
	25	3946.4 (0.24)	3810.0 (1.21)	3984.0 (0.98)	4032.0 (1.22)	4.567	1.205

## Data Availability

Data will be made available on request.
